# 

**Published:** 1890-04-05

**Authors:** 


20 608

### The Hospital

A Weekly Institutional Journal of

### Science, Medicine, Nursing, and Philanthropy.

EDITED BY

### HENRY C. BURDETT,

AUTHOR OF "HOSPITALS AND THE STATE; 'PAY HOSPITALS OF THE WORLD"; "COTTAGE HOSPITALS, GENERAL, FEVER, AND CONVALESCENT WITH FIFTY BEDS AND UNDER: THEIR ORGANISATION, MANAGEMENT, AND WORK"; "BURDETT'S HOSPITAL ANNUAL"; "HELP IN SICKNESS"; "HELPS TO HEALTH"; ETC., ETC.

ACTING EDITOR

### GEORGE W. POTTER, M.D.,

AUTHOR OF PAPERS ON "BACTERIOLOGY"; "SCARLET-FEVER"; "RHEUMATISM"; ETC. ETC.

SURGEON GEN'L'S OFFICE LIBRARY
136666

### INDEX TO VOL. VIII.

(5TH APRIL TO 27TH SEPTEMBER, 1890.)

FOR 1890.

LONDON:
PUBLISHED BY "THE HOSPITAL" (LIMITED), 140, STRAND, W.C.

N069X

### INDEX TO VOL. VIII. 1890.

Abroad and at Home, 289
Abscess of Bone, 395
Accuser, A Skulking, 31, 41
Addison's Disease, 83
Africa, Tropical, Can it be Colonized, 206
Aix-les-Bains, 83
Albuminuria, 219
——— and Life Insurance, 331
Alcohol, Reduction of Hospital Expenditure on, 8
Alcohol, Use of, 179
Alexandra Hospital, The, 9
All Saints' Convalescent Home for Children, 217
Aloes, Use of, 334
Ambulance Service of the Metropolitan Asylums Board, 8
Ambulance Service, London's New, 328
American Medical Journal, An, 363
Aneurism, Popliteal, 40
Antifebrin, Poisoning by, 285
Antipyrin, 348
Antisepsine, 155
Anti-Vivisection Society, 168
Appeals, When Ought They to Cease? 169
April, 28
Aquarium, The Fasting Man at the, 10, 23, 50, 66
Arsenic, Effects of, 221
Arterial Suture, 220
Arterio Venous Aneurism, Cure of by Extirpation, 220
Asiatic Cholera in London, Alleged, 328
Aspiration in Pneumo Thorax, 69
Asylum Attendants, The Hours of, 8
——— Construction, 55
Asylums, Glances at Foreign, 57, 139, 153
Atrophine, in Poisoning by Aconite, 155
August, 278

Bachelors, Tax on, in France, 265
Baden-Baden, 35
Ballantyne, Dr. J. W., on Sclerema and Œdema Neonatorum, 53
Barnardo's Homes, Dr., 153, 178
Benedictine Order, The, and the Pope, 22
Bicêtre Asylum, 22
Birmingham Children's Hospital, The, 15
——— Ear and Throat Hospital, 344
——— Queen's Hospital, 22
——— Hospital Reform Committee, 217, 250
Black Forest, A Hospital in the, 325
Blandford Cottage Hospital, 233
Blind, Conference on the Education of the, 265
Bloomingdale Asylum, New York, 57
Blyth, Mr. Wynter, on the Diphtheria Epidemic, 22
Bone, Reparation of, after Trephining, 364
Bolton Infectious Hospital, 392
Boscombe Hospital, 22
Boston (U.S.A.) Lunatic Hospital, 139
Bournemouth, Herbert Convalescent Home, 361
Bovril, 318
Brewing, 68
Brighton and Hove Dispensary, 36
——— Sussex County Hospital, 31, 41, 67
Bright's Disease, Chronic, 379
Bristol Children's Hospital, 8
——— General Hospital, 281
British Medical Association, 289
——— Benevolent Fund, 154
——— Nurses' Association Nurses' Register, 38
——— Ophthalmic Hospital, Jerusalem, 361
Broadbent, Dr. W. H., on the Pulse, 27, 55
Brookwood Asylum, 352
Buffalo (U.S.A.) General Hospital, 95
——— State Asylum, 139
Burdett, Mr. Henry C., on the Pay System, 47
Burns, The Connection of, with Ulceration of the Duodenum, 26
Burroughes and Wellcome and Stanley's Travels, 249
Butter, Normandy, 202
Buzzard, Dr., on Hysteria, 11

Calcium, Iodate of, as an Antiseptic, 172
Calculus, Renal, 12
Callaway, the late Bishop, 52
Cambridge, Princess Mary of, 96
Canal Boats and their Occupants, 10
Cancer, Cure of, by Erysipelas 285
Cannabis Indica, 112
Canterbury Hospital, 400
Carcinoma of the Larynx, 398
Catarrh, Gastric, 11
Cattle, Tuberculous, 202
Central London Throat and Ear Hospital, 328
Charitable Accounts and the C.O.S., 16
——— Trusts and the Charity Commissioners, 101
Charities, General, 239, 255, 287, 294, 318, 352, 368, 384
Charity Officials, Retiring Allowances to, 59, 86
——— Organization Society and Charity Accounts, 16
——— and House of Lords' Select Committee, 42, 43, 100
———, 297
Chelsea, Brompton, and Belgrave Dispensary, 345
Chelsea Hospital for Women, Convalescent Home, 168
Children and the Country, 186
———, Nocturnal Incontinence in, 267
Children's Country Holidays Fund, 81, 94, 110, 200
Chivalry, A Tale of, 178
Christian City, A, 115
Cholera Scare, The, 346
"City of Paris" Disaster, The, 66
Clapham Maternity Hospital, 264
Clark, Sir Andrew, and the Royal College of Physicians, 52
Cleanliness and Health, 282
Clubbe, Dr. C. P., on Surgical Treatment of Diphtheria, 98
Cocaine, Prevention of the Toxic Effects of, 285
Colotomy, 12
Constipation, 53
Construction of Asylums, The, 55
Consumption, A Step Forward, 321
Cork Hospital for Women and Children, 200
Cornwall Royal Infirmary, 328
Craven, Mrs. Dacre, An Interview with, 308
Crede, Dr., on Cystotomy and Cystectomy, 71
Cremation, 103
Cresswell, Mr. George, on the Diseases of the Ox, 113
Cucumbers, 202
Cumberland Infirmary, 249
Currie, Sir Edmund Hay, on the Provident Principle, 3
Cyclists and Rupture, 314
Cystotomy and Cystectomy, 71
Cysts, the Treatment of, 395

Damien, Father, 94
Darwinism and Miss Layard, 362
Dayton Asylum, Ohio, 57
Degraissage, 68
Denmark and Sweden, Jottings from, 92
Dental Hospital of London, The, 9
Dera Ghazi Khan, Medical Mission to, 36
Derby Convalescent Home, 232
——— County Asylum, 352
——— New Infectious Diseases Hospital at, 109
——— the Earl of, on a Provident Out-Patient Department, 89
Derbyshire, Infectious Diseases Hospital at, 109
——— Infirmary, Sanitary Condition of, 50, 161, 185
Devonshire Hospital, and Buxton Bath Charity, 37, 232
Diabetes, Treatment of, 283, 380
Diarrhœa, 347
——— Tropical, 363
Diet, 241
Diphtheria, Calomel in, 14
——— Epidemic, Mr. Wynter Blyth on, 22
——— Paralysis after, 69
——— Surgical Treatment of, 97
——— The Bacillus of, 204
Diuretin, 155
Doctoring John Chinaman, 46
Doctors, A Parson on, 96
——— and Drink, 82
——— Education and Benevolence, for, 61
——— Latin, Professor Huxley on, 314
Doctors' Pulpit, The:—
Women's Uncongenial Occupations, 5
Cares and Coffins, 19
A Gossip about Pills, 33
For the Complexion, a Rival to Pears, 45
A Dead Journalist, 63
A Doctor on the Stump, 77
Country v. Town Practice, 91
Cremation or Decent Burial, 103
A Physician's Apologia, 147
Those that Travel by Water, 163
The Medical Calf, 179
Mountain or Sea, 195
The Man and the Book, 213
On the March, 227
How Suicides are Made, 245
Baby Brains, 259
Bright's Disease and Life Insurance, 275
Race Rivalries, 291
The Expansion of Medicine, 309
Cholera Prevention, 323
More Life and Fuller, 339
Plucked, 359
Cancer Curers, 371
Commonplace, 387
Do the Sick Fear Death? 76, 136
Douche, a New Nasal, 203
"Dreadnought," The Baby, 181
Dreams, Dr. Julius Nelson on, 37
Dropsy, Cardiac, Calomel in, 157
Drugs, Food, Instruments, &c., 332, 348
Duelling and Development, 378
Dufferin's Fund, Lady, 234
Duncan, The Late Dr. Matthews, 362
Dundee Infirmary, 168
Dunlop, Dr. Andrew, Remarks on Acute Tonsilitis, 25
Durham County Hospital, Waddington Bequest to, 37
Ear Diseases, 381
East End Mothers' Home, 153
Eccles, The Hope Hospital Scandal, 296, 329
Edinburgh Charities, 313
——— Royal Infirmary, 8
Education and Benevolence for Doctors, 61
——— Elementary, 38
Egypt as a Winter Resort, 43
Electric Prostration, 220
Electricity, Execution by, 298
"Elephant Man," The, 37
Elissen, Dr., on Inflammation, 71
——— Pulsating Exophthalmos, 70
Enteric Fever, Recent Views of, 187
Epilepsy, 39
——— and Paralysis, Hospital for, 264
Epileptics, A New Home for, 330
——— in America, 313
Epsom College, 38, 59, 61, 94
Errors, Popular, 38
Ether, Use of, 334
Exalgin, 205
Examination, The National Teachers' Union annual, 38
Example Before Precept, 256
Exophthalmos, Inflammatory, 71
——— Pulsating, 70
Experts, 82

Fairy Tales of Science, 305
Farm Houses for Summer Holidays, 234
Fasting Man, The, 10, 23, 50, 66
Fat, Absorption of, 204
Fawcett, Miss, A Girl of Quality, 154
Fear of Death in Sickness, 76, 136
Felixstowe Convalescent Home, 376
Felixstowe Cottage Hospital, 185
Femur, Unreduced Dislocations of, 236
Fernie, W. T., M.D., on Herbal Simples, 6, 62, 90, 148, 181, 196, 212, 228, 246, 356, 373
——— Influenza and Colds, 49
Finance, Hints from America on Hospital, 4
Fischer, Dr. P., a Rare Case of Symblepharon, 71
Florador, 301
Flower, Mr. Cyril, M.P., 81
Food, Drugs, Instruments, &c., 332, 348
Foreign Asylums, Glances at, 57, 139
Fractured Legs, Treatment of, 301
French Hospital, The New, 216
Friend and Foe, 100
Frivolity's Labours, 304

Gall Stones, The Solution of, 69
Gangrene, 54
Gas Poisoning, 252
Gastrotomy, 318
Germany, Vaccination in, 32
Girls' Home, Forest Hill, 184
Gladesville (N.S.W.) Hospital for the Insane, 139
Gladstone, Mrs. W. E., Appeal by, 170
——— Mr. W. E., On the Position of Physicians, 37
Glances at Foreign Asylums, 57, 139
Glanders, Case of, at University College Hospital, 22
Glands, Mr. Edmund Owen, on Enlarged, 36
Glaucoma in Early Life, 71
Gloucester General Infirmary, 34
Godlee, Mr. Rickman, J., on Examination of the Liver, 53
Goitre, Treated by Electricity, 301
Gould, Mr. A. Pearce, on Work, Play, and Success, 355
Gout, Diet in, 157
Governors, Meetings of, 400
Great Yarmouth Hospital, 216
Grosvenor Hospital for Women, 81
Guinea Worm, 397
Gussenbauer, Dr., on Splenectomy, 70
Guy's Hospital, Opening of a Residential College at, 10

Haeckel, Dr. H., on Phosphorus Necrosis, 70
Hangchow Hospital and Medical Training College, 46
Hausemann, Dr. David, on Paralysis after Diphtheria, 69
Haweis, Rev. H. R., on Doctors, 96
Hawkins-Ambler, Mr. G. A., on Colotomy, 120
Health Resorts, 99
Heart Disease in Children, 347
Heath, Mr. Christopher, on Popliteal Aneurism, 40
Hebrides, Life in the New, 351
Heister, Dr. Lawrence, on the Progress of Surgery, 111
Hemiplegia in Pneumonia of Children, 300
Hepatic Fever, 97
——— Surgery, 220
Herbal Simples, the History and Capabilities of, 6, 62, 90, 148, 181, 196, 212, 228, 246, 356, 373, 390
Hertford Infirmary, 36
Holidays and Health, 320
——— Short Trips for Short, 367
Home Hospitals Association, 47, 51, 169
Homœopathic Hospital, 80
Hop Industry, The, 17
Hops and their Rivals, 68
Hop Tea, 346
Horseflesh as Human Food, 24

Horton Infirmary, 313
Hospital for Sick Children, Great Ormond Street, 9, 168
Hospital Saturday Fund, Metropolitan, 184
—— throughout the Country, 392
—— in Birmingham, 95, 201
—— Brighton, 360
—— Bournemouth, 328
—— Newcastle, 216
—— Warwick, 217
—— Woolwich, 376
Hospital Sunday Fund, Metropolitan, and Hospital Accounts, 281
Hospital Sunday, 115, 135, 140
—— Fund, Metropolitan, 169, 262
—— Pontefract, 344
Hospital Workshop, The:—
Birmingham Children's Hospital, The, 15
Gloucester General Infirmary, 34
London Hospital Hebrew Wards, 175
Stroud General Hospital, 85
Hospitals and Sick Children, 117
—— Association, The, Annual Meeting, 153
—— New Members of the, 50
—— Papers Read Before the, 3, 80
—— Street Ambulance Branch, 50, 80
—— The Provident Principle in Connection with, 3
—— The Story of the, 117
—— Home and Pay, 47
House Surgeon, Stockton-on-Tees Hospital, Salary of, 52
Huddersfield Infirmary, 265
Hume and Miracles, 266
Hutchinson, Mr. Jonathan, on our Hospitals, 145
Huxley, Professor, and Doctors' Latin, 314
Hygiene and Demography, Congress of, 201
—— International, 218
Hypnotism, 23, 24, 188, 282
—— and the Daily Press, 282
Hysteria and Organic Disease, 11, 25

Ill-Health, Thoughts on, 285, 310, 324
Illinois, Southern Hospital for the Insane, 57
Imperial Provision for the Sick, 131
Inebriety, 39
Infirmaries, Origin of, 315
Influenza, Indirect Mortality from, 155
Influenza, "The Times" on, 37
Inquisition, a Hospital, 43
Insane, The Story of, from Year to Year, 352, 377, 393
Insane, Watching of the, by Reflecting Shafts, 78
Insanity cured by Influenza, 203
Insect Stings, 300
Instruments, Food, Drugs, &c., 332, 348
Invalid Children's Aid Association, 184
—— Furniture, 302
Ipswich Hospital, 139

Jacobson, Mr., on Renal Calculus, 12
Jews and Athletics, 171
"John Chinaman," Doctoring, 46
Jottings from Sweden and Denmark, 92
July, 207
June, 159

Kaffraria, The late Bishop of, 52
Kensington, The Health of, 265
Kent Christians, 378
Kilmarnock Infirmary, 249
King's College Hospital, 37
Kissing, The Dangers of, 20
Kleptomania, 346
Knowledges, The Two, 354
Knuke, Dr. H., Guide to the Demonstration of Bacteria in the Tissues, 48
Kyrle Society, 234

Laborde, Dr., on Sodium v. Potassium Salts, 71
Lady Guardians of the Poor, 81
Lady Novelists and Tall Men, 266
*Lancet*, The, and Hospital Nurses, 264, 288
Lanolin, 285
Laqueur, Dr., Glaucoma in Early Life, 71
Laryngeal Disease, Sudden Deaths in, 236
Laryngitis, Traumatic, 39
Law, Mr. John, "A Manchester Shirt Maker," 49
Layard, Miss, and Darwinism, 362
Lees-Knowles, M.E., Mr., on Horseflesh, 24
Leprosy, Dr. Beaven Rake, on, 317
—— Fund, The National, 94
—— Investigation, 382
—— Recent Views of, 251
Life, A Future, and Science, 17
Lilly, Mr. W. S., and Mr. Herbert Spencer, 24
Lister, Sir Joseph, and the Spray, 314
Liver, Examination of the, 53
Liverpool Infirmary, 153
Loch, Mr. C. S., on Medical Relief in London, 42
Longe Point Asylum, 94
London Charities, The Year's Work of the, 116, 141
London Hospital, Attack on the Matron of, 345
—— and the Lords' Commission, 258, 345
—— Charges Against It, 369
—— Life in a, 255, 276
—— Officials of the, 320
—— Sanitary Condition of the, 28
—— The, 109
London, Medical Relief in, 42
—— Post Graduate Course, 114
London's New Street Ambulance Service, 328
Lords, Select Committee of the House of, on Metropolitan Charities, 43, 51, 67, 100, 105, 133, 149, 165, 181, 197, 229, 243, 257, 261, 272, 277
Louth Hospital and Dispensary, 264
Lucknow, Opium Eating at, 23
Lunacy Laws, The New, 223
Lunacy Statistics, 250
Lunatics, Dangerous, at Large, 336
Lungs, Resection of, 204

Madden, Dr. T. More, on the Chronic Diseases of Women, 68
Malarious Hæmophylia, 235
Mamma, Excision of the, under Hypnotic Anæsthesia, 365
Manchester Royal Infirmary, Alleged Mismanagement at, 36
—— Royal Infirmary, 297
—— The Health of, 313
Maple, Mr. Blundell, 81
Margate Royal Sea Bathing Infirmary, 249, 280, 297
Marlborough, Proposed Infectious Diseases Hospital at, 81
Marley, Dr. T., on Tracheotomy, 39
Marriage, is it a Sin? 225
Mary Wardell Convalescent Home, 108
Mattei, Count, the Cancer Curer, 140
May, 64
—— and Missionaries, 96
—— in India, 114
McClachan, Dr. J., on Applied Anatomy, 84
Meat Extracts, the Value of, 249
—— Poisoning by Bad, 69
Medical Charities of London, A Year's Work in the, 116, 141
—— Degrees for Women, 185
—— Institutions of London, 315
—— Officers and their Qualifications, 87
—— Relief in London, 42
—— Term, A New, 244
Medicine, Free Trade in, 337
—— Scientific, an Endless Quest, 385
—— The Gateways of, 357
—— The Public Aspects of, 273
Melbourne, Alfred Hospital, 376
Mesmerism at the Westminster Aquarium, 191
Metropolitan Asylums Board, The, and their Hospitals, 131, 312
Metropolitan Charities, Select Committee of House of Lords on, 42, 43, 51, 67, 100, 105, 133, 149, 165, 181, 197, 229
—— The Year's Work of the, 116
—— Hospital, The, 81, 89
Middlesbrough, North Riding Infirmary, 37
Middlesex Hospital, The, 95
Midwives of Old, 166
Mildmay Home, 94
Military Exhibition, The, 137
Milk, The Dangers of Tuberculous, 330
Miller Hospital, The, 344
Miracle, A Modern, 194
Mission, Medical, to the Dera Ghazi Khan, 36
Morphine, A New Antidote for, 155
Myxœdema, 111

Nasmyth, The Bequests of Mr. James, 312
National Conference of Charities and Correction, The, 36
—— Health Society, 95
—— Leprosy Fund, 94
Necrosis, Phosphorus, 70
Nelson, Dr. Julius, on Dreams, 37
Nephrolithotomy, 267
Neuralgia, Injections of Ether in, 284
Newcastle, New Infirmary for, 36
New Gallery, Exhibits at the, 80
New Hospital for Women, The, 67, 72, 94
New Hospitals, 66, 80, 81, 109
New York, Bloomingdale Asylum, 57
"Nona," the New Disease, 14
Norfolk and Norwich Hospital, A Convalescent Home for, 201
Northampton (Mass.) Lunatic Hospital, 139
North-Eastern Hospital for Children, 36, 152
North London Consumption Hospital, 81, 168
North Riding Infirmary, Middlesbrough, 37
North-West London Hospital, 168
Norwich City Asylum, 352
Nothnagel, Professor H., on Addison's Disease, 83
Nottingham Children's Hospital, 94
Nurses' Holidays and Rests, 110
Nurses, The Wrongs of Private, 298

Oban Cottage Hospital, 296
Oedema Glottidis as a Result of Iodism, 203
Officials, Charity, Retiring Allowances to, 59, 86
—— of the London Hospital, 320
O'Hardon, Dr Virgil, on Pelvic Inflammation, 83
O'Neil, Dr., on Gangrene, 53
Ohio, Dayton Asylum, 57
Opium-eating at Lucknow, 23
Optic Neuritis, 396
Ord, Dr. W., on Myxœdema, 111
Orphanhood, The Prevention of, 145
Out-patients, A Question about, 384
—— in Hospitals, 399
Ovariotomy, 237
Owen, Mr. Edmund, on Enlarged Glands, 26
Ox, Diseases and Disorders of the, 113

Paddington Board of Guardians, Resolution of, 315
—— Diphtheria at, 297
Paget, Sir James, on Education and Benevolence for Doctors, 61
Paisley Sick Hospital, 185
Paracresotine Acid, 158
Paralysed and Epileptic, National Hospital for, 264
Paralysis after Diphtheria, 69
Parke, Surgeon, 108, 201
Parson, A, on Doctors, 96
Pasteurism and Politeness, 266
Pay System in Hospitals, Mr. Henry C. Burdett on the, 47
Pekin, London Missionary Hospital at, 392
Pelvic Inflammation, 83
Pensions to Charity Officials, 58
Pepper, Dr. W., on Hepatic Fever, 97
Perth Royal Asylum, 55
Philadelphia Croup Ward at Children's Hospital, 8
Phosphorus in Rickets, 155
Phthisis, Hot Air Treatment of, 219
—— Nature of, 235
Physicians, The Royal College of, 52
Pleurisy, Salicylates in, 68
Pneumothorax, Aspiration in, 69
Pneumotomy, 70
Podophyllin Poisoning, 221
Poisoning by Bad Meat, 69
Politicians and Physicians, 10
Pomroy, Dr., on Epilepsy, 39
Pope, The, and the Benedictine Order, 22
Popliteal Aneurysm, 40
Popular Errors, 38
Post-Graduate Course, The London, 114
Potassium Salts v. Sodium, 71
Poulton, Mr. F. T., on "Social Environment," 23
Powders in Papers, 221
Prescriptions in the Mother Tongue, 82
Princess, The, and the First Thousand Nurses, 209
Procession, A Terrible, 218
Progress of Surgery, 111
Property, Private, The Moral Basis of, 292
Provident Out-patient Departments, 89
—— Principle in connection with Hospitals, The, 3
Pruritus Neurotic, 69
Psilosis, 299
Pugilists and Bulldogs, 394

Qualification Question, The, 87
Quebec Lunatic Asylum, 57
Queen's Hospital, Birmingham, The, 22
Quinine, A Substitute for, 221

Ragged School Union, 96
Rake, Dr. Beaven, on Leprosy, 317
Ramsay, Dr., on Pneumotomy, 70
Reading, Royal Berks Hospital, 36
Reflecting Shafts for Watching the Insane, 78
Relief, Medical, in London, 42
Religion, Wanted a, 186
Research, Recent, 267, 380
Resorcin, 255
Retiring Allowances to Charity Officials, 59, 86
Reviews:—
A City Girl—John Law, 164
A Manchester Shirt Maker, 49
Anatomy—Edmund Owen, 287
Applied Anatomy, Medical, Surgical, and Operative, 84
Bayley's Pocket Book for Pharmacists, 334
Browning's Message to His Time—Edward Berdoe, 198
Chambers' Encyclopædia, 294
Egypt as a Winter Resort, 48
Essentials of Forensic Medicine—G. A. Semple, 349
Faith Cures: Their History and Mystery—A. J. L. Gliddon, 260
Guide to the Demonstration of Bacteria in the Tissues, 48
Harvey on the Circulation of the Blood—Dr. A. Bowie, 269
Home Medicine and Surgery—Dr. W. J. Mackenzie, 326
Influenza and Colds, 49
Leçons de Gynécologie Opératoire, 113
London Medical Specialists—C. B. Allen, 254
The Medical Annual, 14
The Natural History of Specific Diseases, 14
The Psychology of Attention—M. Ribot, 190
The Pulse, 27, 55
Twelve English Statesmen, 150
What Cheer O?—A. Gordon, 326
Reynolds, Dr. J. Russell, on the Toxic Effects of Indian Hemp, 112
Right on Left, 210
Roads, Country, 1
Romford Cottage Hospital, 169
Royal Hospital for Incurables, 94
Royal Portsmouth Hospital, 81
Russia: Its People and Affairs, 78
Ryan, Mr. Thomas, on London's New Street Ambulance Service, 80

St. Mary's Hospital, 184
Salaries of House Surgeons, The, 52
Salicylates, 156
Salisbury Infirmary, 361

134666

Samaritan Free Hospital, 67, 94
Sandwith, Mr. F. M., F.R.C.S., on Egypt as a Winter Resort, 48
Sanitary Condition of the London Hospital, 30
——— Congress, 344
——— Institute, 168
——— Reform at Sheffield, 110
Sanity, A Standard of, 73
Santonin, Administration of, 172
Saundby, Dr. R., on Gastric Catarrh, 11
Sausages and Polonies, 24
Savernake Hospital, 22
Science and a Future Life, 17
Sclerema and Œdema Neonatorum, 53
Seamen's Hospital, 233
Seaside Convalescent Homes, West of Scotland, 360
September, 341
Sheffield and Its Sanitary Arrangements, 110
——— General Infirmary, 360
——— Public Hospital, 281
Shipston-on-Stour, Proposed Hospital at, 80
Shrewsbury Dispensary, 23
Sick Children and Hospitals, 117
Skin Grafting, 171
Skull, Temporary Resection of, 156
Slave Trade, Cardinal Lavigerie, and the, 394
"Social Environment," M. F. T. Poulton on, 23
Sodium v. Potassium Salts, 71
Southam, Mr., on the Connection of Burns with Ulcration of the Duodenum, 26
Southport Children's Sanatorium, 281
Spas, German and French, 35
Specialization, Over, 186
Specific Diseases, Dr. E. F. Willoughby on the Natural History of, 14
Spelthorne Sanatorium, 239
Spencer, Mr. Herbert, and Mr. W. S. Lilly, 24
Spina Bifida, 252
Splenectomy, 70
Spray, The, and Sir Joseph Lister, 314
Sprue, 299
State Homœopathic Asylum, New York (U.S.A.), 139
——— Logic, 218
——— Lunatic Hospital, Harrisburg (Penn.), 139
Stiller, Dr., on Salicylates in Pleurisy, 69
——— the Solution of Gall Stones, 69
Stockton-on-Tees Hospital, 52
Stockton Surgical Hospital, 360
Story of the Hospitals, The, 117
Stroud General Hospital, 85
——— Hospital Centenary Celebration, 392
Sublimate Solutions, Advantage of Warm, 204
Succi, Sig. Giovanni, "The Fasting Man," 10, 23, 50, 66
Suicides, Boy, and Doctors, 394
Sulphonal in Diabetes, 205
Summer Duty, A, 110
Sunday Demonstrations, Parades, and Processions, 281, 297
Sunderland Infirmary, 280, 392
Sunflower, The, as an Antiperiodic, 364
Surgery, Sympathetic, 374, 389
Surgical Treatment of Diphtheria, 97
Sussex County Hospital: Refutation of the Allegations Against, 31, 41, 67
Swansea Eye Hospital, 265
Sweden and Denmark, Jottings from, 92
Symblepharon, A Rare Case of, 71

Tabes, Nitrate of Silver in, 203
Table Springing, 24
Tait, Dr. Lawson, on Typhlitis, 25
Tea, A New, 343
Teachers' National Union, 38
Tetanus, Traumatic, Treatment of, 156
Tewkesbury Rural Hospital, 280
Theory and Practice, 354
Therapeutics, Recent, 365
Thomas, Dr., on Constipation, 53
Tissues, Demonstration of Bacteria in the, 43
Tonsilitis, Acute, 25
Tracheotomy, 39
Traumatic Laryngitis, 39
Trephining, Restoration of Bone after, 284
Trivialities, 272
Tyler, Bequests by the late Sir James, 50
Typhlitis, 25
Typhoid, The Bacillus of, 13

University College Hospital, 8
——— Case of Glanders at, 22
——— Degrees and Honorary Medical Officers, 87
Unqualified Assistants, 298
Unsectarian Hospitals, 140
Ureametry, 235
Vaccination in Germany, 32
——— Italy, 84
Varicose Veins, The Injection of, 204
Vegetarianism, 241
Vegetarians, What They Want to Know, 378
Venesection, 283
Vesical Tumours, 397
Victoria Hospital for Children, 169

"Wake up Mary," 96
Wales, A Medical School for, 250
Wales, Prince and Princess of, in Southwark, 265
Wallace, the Late Sir Richard, 250
Walsall Cottage Hospital, 185
Watching the Insane by Reflecting Shafts, 78
Wertheimer, Dr., on Neurotic Pruritus, 69
West Bromwich District Hospital, 280, 312
West Ham Hospital, 66
What is an Expert? 82
Whitehaven and West Cumberland Infirmary, 36
Willis, Mr. Armstrong, on Inebriety, 39
Willoughby, Dr. E. F., on the Natural History of Specific Diseases, 14
Women, Brain Fag in, 170
——— Chronic Diseases of, 68, 90
——— German and English, 362
Wood, Dr. H. C., on Hysteria, 25
Woolwich Cottage Hospital, 264
Words of Consolation:—
Care, 306
Come, 355
Failure, 338
Death, 32
God's Hand, 174
God's Ministers, 383
Helps to Gain Patience, 88
Hope, 194
Kindness Shown in Suffering, 242
Patience, 60; Patience in Sickness, 74
Prayer, 178
Prayer for Recovery, 226
Reading the Scriptures, 146
Redemption, 162
Salvation, 370
Submission, 258
The Burthen of Life, 210
The Lord is Risen, 2
The Power of Pain, 44
Truth, 18
Unbelief, 290
Wasted Opportunities, 322
Wearisome Days, 132
Weary Nights, 102
Work, Play, and Success, 355
Working Women in Large Towns:—
Domestic Servants, 342, 372
Factory and Workroom Hands, 175
Factory Girls, 104
In England and in America, 56
Matchbox, Brush, and Box Makers, 293
Needlewomen, 214
Shirt, Sack, and Umbrella Makers, 335
Shop Assistants, 388
Street Workers, 75
Women as Tailors, 182
Workhouse Infirmaries, The Neglect of the Poor in, 270

Year's Work of the Medical Charities of London, The, 116, 141
Young Women's Christian Association, Educational Committee, 392

Zenana Bible and Medical Mission, 185
Zurich University, A Visit to the Hospital at, 27

### THE SPECIAL NURSING SUPPLEMENT (MIRROR).

Abdominal Diseases, ii
Acland, Sir Henry, on Nursing, ix
American Doctors and Nurses, xvii
——— Note, An, lxxii
——— Notes from, lxxix
——— Nurses' Directories, xci
——— Nursing in, ciii
——— Papers, xvii
——— Training Schools for Nurses, xxxiii
Appointments, iv, vii, xii, xiv
Asylum Attendants, lxxxiii, xcix
——— Their Work and Training, xlii
Asylum Nurses, xv
Atkinson, Dr., on Infection, xxi
A Turn of the Wheel, ci
Australia, Hospital Commission in, lxxxvii, xcvi
——— Notes from, liv, lxxiii
Beef Tea, xiii, lxv
British Nurses' Association, lxxv
——— Register, i, xiii, xvii, xxix, xxxviii
——— Why Hospital Authorities Object to the, xciii
Catholic Nurses, xcvi
Chicago, Nursing in, i
District Nursing, i, v
——— A History of, W. Rathbone, M.P., xlvi
Doctors and Nurses, American, xvii
Doctor Tarrant's Mistake, lxxvi, lxxx, lxxxiv
Dying, Nurses' Duties to the, xcv
Epileptics, Homes for, lxxvii
Examination Questions, iv, xxiii, xxvii
Feeding, x
Fever Nurses, lv
Glasgow Medical School for Women, cvii
Hospital Commission, The, lxvii.
——— Nurses, cii
——— The Working Hours of, lxix
——— Six Months in a, cix
Insanity, Sketches of, xlvi, lxiii, lxviii, lxxii, lxxvi, lxxx, lxxxviii, xcii, xcvi, c, civ, cviii
Insomnia, xli
Institutions, Nursing, i, v, ix, xiii, xvii, xxi, xxv, xxix, xxx, xxxiii, xxxvii
Johannesburg, Life and High Life at, lxxxv
Kalihi, News from, xiii.
Kent, Nursing Institution, lxxv
Kidneys, The, xiv
"Lady" Nurses and Domestic Servants, xcvii, cvi
Lectures, Nursing, ii, viii, x, xiv, xviii
Leper Hospital at Robben Island, The New, xvii
Lunatics, The Charge of Private, xxvii
Lewis Percy, M.D., Lectures by, ii, x, xiv, xviii, xxii, xxvi
London Hospital, Nursing at the, lxxxvi
——— The attack on the, xciv
Love, vii
Loyalty, i
Luff, Dr. A. P., on Beef Tea, xiii
Male Nurses, lxxxvii
Manchester, District Nursing at, xli
Massage, lxxx
Matron and House Surgeon, xlv
Matrons, Trained, xlix
Melbourne, a Strike among Nurses at, lxxxiii
Meum and Tuum, iii
Midwives and Monthly Nurses, The Training of, xxix
——— Registration of, xxxiii, xliii
——— Trained, cii
Morgan, Memoir of the late Junius S., vi; Suggestion for a Memorial to, ix, xv, xix
Nightingale Nurses, lxvii
Notes and Queries, iv, viii, xii, xvi, xx
Nurse Joyce, xxx, xxxix
——— in a Lodging-house, A, xlvi
——— in Trouble, A, xci
——— Training School, xii
——— the Work of the Private, xcv
Nurse's Holiday, a Catholic, cviii
Nurses, a Home of Rest for, xxxiii, xliii, lv, lxxiii
——— Anæmic Look of, xliii
——— An Appeal to, xcix
——— and Doctors, American, xvii
——— and Infection, xxi
——— a Novel for, xcvii
——— are they Sweated, cv
——— Asylum, xv
——— Catholic, xcv
——— Excursions for, xlv
——— Home, Bucks, lvii
——— Johannesburg, lxxxvii
——— Sheffield, liii
——— in Asylums, lxxvii
——— Intemperate, xc
——— Ladies as, lvii
——— Male, xvi
——— Missionary Association, xvii
——— Mutual Society, iv, xxii
——— Newspapers, American, xvii
——— on their Travels, civ
Nurses, Queen Victoria's Jubilee Institute for, lx, lxii
——— Sir Henry Acland on, ix
——— The Registration of, lxix
——— The Training of, in France, liii, lxxii, lxxxix
——— The Working Hours of Hospital, lxix, lxxv, xcvii
——— Trained Male, lxxi, lxxxvii
——— Wanted, lxxi
Nursing as a Profession, civ
——— Details, Noble Lords on, lxxi
——— District, i
——— Institutions, i, v, ix, xiii, xvii, xxi, xxv, xxix, xxx, xxxiii, xx vii
——— Lectures—
VII. Abdominal Diseases, ii
VIII. Feeding, x
IX. The Kidneys, xiv
X. The Skin, xviii
XI. Nervous Diseases, xxii
XII. Conclusion, xxvi
Nursing Uniforms, Exhibition of, i
Outrage, a Daring, at the Seamen's Hospita lxxxix
Papers, American Nurses', xvii
Paris, Nursing in, ix
Pension Fund for Nurses, The Fête of the First Thousand, l, lvii, lviii
——— The First Thousand, vii, xii, xv, xvii, xx, xxii, xxix
——— The National, i, v, ix xli, l, lvi
Philadelphia, liii
Queen Victoria's Jubilee Institute, xix, lxii
Queen Charlotte's Lying-in Hospital, xxix
Robben Island Leper Hospital, xvii
Rural Nursing Association, cvii
Skin, The, xviii
South Africa, A Letter from, xxxv
Southsea Home, vii
"Sweating" among Nurses, cv
Tasmania, News from, iii
The Passing of the Shadow, xi, xv, xix
Uniforms, Exhibition of Nursing, i
——— for June, vii, xvii
Wales, H.R.H. the Princess of, Presentation to "The First Thousand" by, vii, xii, xv, xvii, xx, xxii, xx[illegible]
Winchester Hospital, xlix
Workhouse Nurses, lxvii, lxxxi, lxxxix
Zenana Medical College, lxxxvii

### NEW OFFICES IN THE STRAND.

The Hospital *is now published at* **140, Strand, W.C.** *It has been found desirable to establish* The Hospital *in this central and convenient locality, owing to the rapid extension of the business of this paper of late.*

### NOTICE TO CORRESPONDENTS.

All MS., Letters, Books for Review, and other matters intended for the Editor should be addressed The Editor, The Lodge, Porchester Square, London, W.

**Notice to Advertisers and Subscribers.**

All Advertisements, Orders for Copies of The Hospital, and Business Communications should be addressed to The Publisher, *not to the Editor*, The Hospital, 140, Strand, W.C.

**Bound Volumes of "The Hospital."**

**Vol. VI.**, containing full page portraits of T.R.H. the Prince and Princess of Wales, and Miss Florence Nightingale, is now ready, 4s. post free. Cases for binding may be had of the Publisher, at 1s. 6d. each.

**To Residents, Secretaries, and Matrons.**

The Editor will be glad to have the Regulations and each Report as issued by Asylums, Hospitals, Convalescent and Nursing Institutions, as well as those of other Charities, and an early copy in duplicate of all Appeals and Festival Notices, etc., addressed to *The Editor, The Lodge, Porchester Square, London, W.* Any superintendent, secretary, matron, or other chief official who regularly sends such information may be registered, on application to the Publisher, to receive free of cost a cover for binding The Hospital in half-yearly volumes.

### Scraps and Gleanings.

The Queen has sent a quantity of cast linen to the Cancer Hospital, Brompton.

Coventry Workhouse Infirmary was opened by Mr. Beamish with a silver key.

Sunderland Provident Dispensary is in a sound financial position, and is self-supporting.

Mr. Robinson, once house surgeon, has been elected honorary surgeon to Doncaster Infirmary.

Monkstown Hospital treated 250 in-patients last year. The building fund now exceeds £2,000.

Aberdeen Sick Children's Hospital treated 500 cases last year, of which 50 were infectious cases.

Watlington Cottage Hospital reports an increase in expenditure, but also an increase in subscribers.

It is stated that the late Duke of Manchester has left directions in his will for his remains to be cremated.

Llandrindod Wells Cottage Hospital treated 135 patients last year. Average cost per occupied bed, £50 5s.

The Pope has forbidden Catholics to cremate their dead; once more science and religion are in antagonism.

Dr. Woodhead has been presented with a testimonial on the occasion of his removing from Edinburgh to London.

The late Mr. Justice Manisty has left by his will £250 each to St. Mary's Hospital, Paddington, and the London Fever Hospital.

Sir William Jenner is (*Vanity Fair* hears) about to retire shortly from the active exercise of his profession, and to establish himself at Southsea.

Mr. Arthur E. Ronald, M.B. and B.S. Cantab, and M.R.C.S., has been appointed junior resident medical officer to the Royal Free Hospital.

The new rules of Bristol General Hospital have been confirmed by a large majority of votes. Dr. Marshall, Dr. Burder, and Dr. Swayne voted in the minority.

Owing to the almost constant prevalence of small-pox at the Reunion Island, it has been seriously considered whether vaccination should not be made compulsory in that island.

The Society for the Study of Inebriety gave a reception to Dr. F. R. Lees, on Tuesday, on the occasion of his coming to reside near London. Dr. Norman Kerr delivered the annual address.

Prince Albert Victor laid the foundation-stone of the Leper Asylum at Bombay on March 25th. Sir Dinshaw Manockjee Petit has given a lakh of rupees towards the cost of the building.

The Victoria Hospital for Children, Chelsea, has sent forth a pretty Easter card bearing the words, "Risen with healing in His wings." The hospital sadly needs the Easter offerings of the rich.

Influenza has made its appearance on board the English ships in the harbour of Zanzibar. It has also broken out in Melbourne, as well as in New Zealand, the malady being identical in character with the epidemic in Europe.

The number of paupers in the metropolis on the last day of the second week of March was 99,138, including 60,733 indoor, and 38,405 outdoor. The number in the corresponding period of 1889 was 105,199; in 1888, 112,747; and in 1887, 103,815.

In the Army Estimates for the year 1890-91 the sum of £294,800 appears under the vote to defray the expense of the pay, &c., of the medical establishment and cost of medicines, &c., being a decrease of £3,700 as compared with the preceding year.

Mr. John Aird, M.P., presiding last week at the annual dinner of the Royal Albert Orphan Asylum, given at the Savoy Hotel, stated, amid cheers, that the Queen had promised an annual subscription of ten guineas to the funds of the charity. Subscriptions were announced amounting to £2,300, including 100 guineas from the chairman.

The Grantham District Coroner inquired into the death of Martha Charles, aged six months, at Stoke, Lincolnshire. The evidence showed that the child was left in the cradle by her mother for a short time whilst she was engaged upstairs. On returning, Mrs. Charles found the cat curled up asleep on the face of the child, the latter being quite dead. A verdict of accidental suffocation was returned.

